# Supplementary material for: Cost-benefit analysis for invasive species control: the case of greater Canada goose Branta canadensis in Flanders (northern Belgium)
Source: PeerJ. 2018 Jan 29;6:e4283. doi: 10.7717/peerj.4283 (PMC5793711; doi:10.7717/peerj.4283)
Supplement: Table S1 — Present value (PV, M€) calculations for the management cost (MC) and damage costs (DC) for BAU and alternative scenarios with an increase in the parameters of the population model (K or r) by 10% and a scenario with a 90% decrease in the price of hay. [file peerj-06-4283-s003.docx]

Table S1. Present value (PV, M€) calculations for the management cost (MC) and damage costs (DC) for BAU and the scenarios with an increase in the parameters of the population model ($K$ or $r$) by 10%. Also a scenario for a sharp decrease of 90 in the price of hay is calculated.

|  |  |  | $\boldsymbol{K}\boldsymbol{+}\boldsymbol{10}\boldsymbol{\%}$ | |  |  | $\boldsymbol{r}\boldsymbol{+}\boldsymbol{10}\boldsymbol{\%}$ | |  | **Price of hay – 90%** | | |
| --- | --- | --- | --- | --- | --- | --- | --- | --- | --- | --- | --- | --- |
| **TYPE OF COST** |  | **BAU** | **Enhanced** | **Δ PV** |  | **BAU** | **Enhanced** | **Δ PV** |  | **BAU** | **Enhanced** | **Δ PV** |
| **PV Damage Costs (DC)** |  |  |  |  |  |  |  |  |  |  |  |  |
| Agriculture |  | 26.46 | 2.59 | 23.87 |  | 24.06 | 2.50 | 21.55 |  | 2.41 | 0.24 | 2.17 |
| Eutrophication |  |  |  |  |  |  |  |  |  |  |  |  |
| Unit cost of N and P (low)* |  | 3.56 | 0.35 | 3.21 |  | 3.24 | 0.34 | 2.90 |  | 3.24 | 0.32 | 2.92 |
| Unit cost of N and P (high, low)* |  | 18.11 | 1.77 | 16.33 |  | 16.46 | 1.71 | 14.75 |  | 16.46 | 1.61 | 14.85 |
|  |  |  |  |  |  |  |  |  |  |  |  |  |
| **PV Management Costs (MC)** |  | 0.00 | 0.28 | -0.28 |  | 0.00 | 0.28 | -0.28 |  | 0.00 | 0.25 | -0.25 |
| **PV DC + PV MC** |  |  |  |  |  |  |  |  |  |  |  |  |
| Eutrophication |  |  |  |  |  |  |  |  |  |  |  |  |
| Unit cost of N and P (low) |  | 30.02 | 3.22 | 26.80 |  | 27.30 | 3.12 | 24.18 |  | 5.64 | 0.80 | 4.84 |
| Unit cost of N and P (high) |  | 44.57 | 4.64 | 39.92 |  | 40.52 | 4.49 | 36.03 |  | 18.87 | 2.10 | 16.77 |

*High - low unit costs (2014 prices) for N: 5.4 - 79.94 /kg and low for P: 86.42 /kg
