# Supplementary figures and images for: Cost-benefit analysis for invasive species control: the case of greater Canada goose Branta canadensis in Flanders (northern Belgium)
Source: PeerJ. 2018 Jan 29;6:e4283. doi: 10.7717/peerj.4283 (PMC5793711; doi:10.7717/peerj.4283)

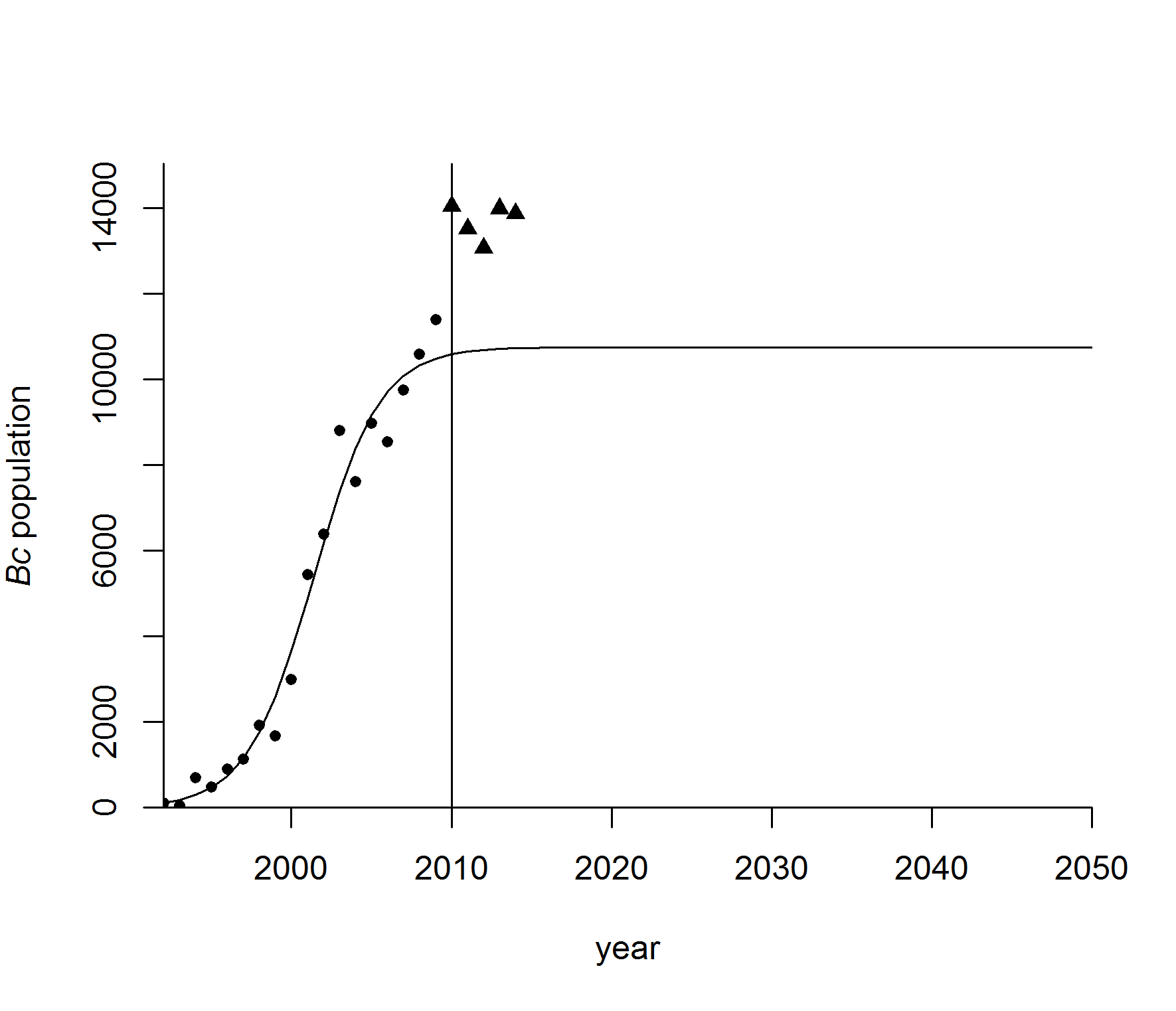

Supplement: Supplemental Information 1 — R code used for the production of figures for Reyns et al. cost-benefit analysis of Branta canadensis in Flanders. [file peerj-06-4283-s004.zip › peerj-21161-Reyns_etal_Rcode/Fig3.tif]
